# Pharmacological and genetic inhibition of fatty acid‐binding protein 4 alleviated cisplatin‐induced acute kidney injury

**DOI:** 10.1111/jcmm.14512

**Published:** 2019-07-08

**Authors:** Zhouke Tan, Fan Guo, Zhuo Huang, Zijing Xia, Jing Liu, Sibei Tao, Lingzhi Li, Yuying Feng, Xiaoyan Du, Liang Ma, Ping Fu

**Affiliations:** ^1^ National Clinical Research Center for Geriatrics and Division of Nephrology, Kidney Research Institute West China Hospital of Sichuan University Chengdu China; ^2^ Division of Nephrology ZunYi Medical University Affiliated Hospital ZunYi China; ^3^ Division of Pharmacy West China Hospital of Sichuan University Chengdu China

**Keywords:** acute kidney injury, apoptosis, cisplatin, endoplasmic reticulum stress, fatty acid‐binding protein 4

## Abstract

Fatty acid‐binding protein 4 (FABP4) has been confirmed to be involved in the pathogenesis of ischaemia/reperfusion‐ and rhabdomyolysis‐induced acute kidney injury (AKI), and targeting inhibition of FABP4 might be a potential strategy for AKI. Cisplatin as a commonly used cancer chemotherapeutic drug possessed a dose‐limited side effect of nephrotoxicity. However, whether FABP4 inhibition exerted a favourable renoprotection against cisplatin‐induced AKI and the involved mechanisms remained unknown. In the study, cisplatin‐injected mice developed severe AKI symptom as indicated by renal dysfunction and pathological changes, companied by the high expression of FABP4 in tubular epithelial cells. Selective inhibition of FABP4 by BMS309403 at 40 mg/kg/d for 3 days and genetic knockout of FABP4 significantly attenuated the serum creatinine, blood urea nitrogen level and renal tubular damage. Mechanistically, cisplatin injection induced the increased apoptosis and regulated the corresponding protein expression of BCL‐2, BCL‐XL, BAX, cleaved caspase 3 and caspase 12 in the injured kidney tissues. Cisplatin also triggered multiple signal mediators of endoplasmic reticulum (ER) stress including double‐stranded RNA‐activated protein kinase‐like ER kinase, activating transcription factor‐6 and inositol‐requiring enzyme‐1 pathway, as well as CHOP, GRP78 and p‐JNK proteins in the kidneys. Oral administration of BMS309403 significantly reduced the number of renal TUNEL‐positive apoptotic cells. Knockout of FABP4 and BMS309403 notably improved ER stress‐related apoptotic responses. In summary, pharmacological and genetic inhibition of FABP4 modulated apoptosis via the inactivation of ER stress in the tubular epithelial cells of cisplatin‐induced AKI.

## 1 INTRODUCTION

Acute kidney injury (AKI), characterized by a rapid decline of glomerular filtration rate, is a clinical syndrome correlated with a high mortality and the increased risk of chronic kidney diseases (CKD).^1^, ^2^, ^3^ Cisplatin, as a commonly used tumour chemotherapeutic drug, possessed a dose‐limited side effect of nephrotoxicity. Cisplatin must be withheld from patients because of potential confounding factors predisposing them to an increased risk for kidney injury, including smoking, hypoalbuminemia, advanced age or known CKD.^4^ Approximately 30% of cisplatin‐administered patients suffered from renal dysfunction and injury, especially AKI.^5^ Unfortunately, treatment strategy for cisplatin‐induced kidney diseases remains limited.^6^

Although the involved mechanisms have not been fully explored, the endoplasmic reticulum (ER) stress‐mediated apoptosis played crucial roles in cisplatin‐induced AKI.^7^, ^8^ ER stress emerged as a key pathophysiological paradigm underlying apoptosis. The presence of misfolded proteins and stresses led to the activation of an adaptive program in the ER, which also known as unfolded protein response (UPR), to restore protein‐folding homoeostasis.^9^, ^10^, ^11^ Initiation of canonical UPR engaged three key signalling pathways, which were mediated by inositol‐requiring enzyme‐1 (IRE1), double‐stranded RNA‐activated protein kinase‐like ER kinase (PERK) and activating transcription factor‐6 (ATF6). The UPR was also linked to the activation of stress kinases such as the c‐Jun N‐terminal kinase (JNK) and splicing of X‐box binding protein 1 (XBP1). In the physiological conditions, signal proteins of ER stress (PERK, IRE1, ATF6) were bound by ER chaperone GRP78 and maintained inactive state. Under ER stress, these ER proteins were dissociated from GRP78 and then activated signal transduction to inhibit protein translation, up‐regulate molecular chaperones to enhance protein‐folding capacity, and/or promote unfolded or misfolded proteins degradation by ER‐related protein degradation (ERAD) pathway. Functionally, depending on the severity and duration of ER stress, UPR could be adaptive or trigger cell death. When cells under excessive or prolonged ER stress failed to resolve the protein‐folding defect and restore homoeostasis in the ER via adaptive UPR pathway, apoptosis was triggered.^12^, ^13^, ^14^, ^15^

Fatty acid‐binding protein 4 (FABP4), as a lipid‐binding chaperone, is highly expressed in adipocytes and macrophages.^16^, ^17^ The mitigation of ER stress and apoptosis is protective effects against atherosclerosis because FABP4 was an obligatory intermediate for macrophage ER stress response to lipids.^18^, ^19^ Chemical or genetic inhibition of FABP4 could improve atherosclerosis and type 2 diabetes mellitus, and targeting FABP4 offered therapeutic for inflammation and metabolic diseases. Previous studies also confirmed that circulating FABP4 depended on renal function in AKI and CKD patients.^20^, ^21^ Urinary FABP4 could predict yearly decline of renal function and could be a novel marker of kidney damage. FABP4 has reported to be potential for pre‐clinical application as a biomarker of drug‐induced kidney injury, and cisplatin had no effect on the urinary and serum levels of FABP4.^22^ However, the function and mechanism of kidney‐expressed FABP4 in cisplatin‐induced AKI remained unknown.

In our previous study, we found that FABP4 contributed to the pathogenesis of ischaemia/reperfusion‐ and rhabdomyolysis‐induced AKI and suggested that FABP4 inhibition might be a promising therapeutic strategy for AKI.^23^, ^24^ In the study, we aimed to explore whether pharmacological and genetic inhibition of FABP4 alleviated cisplatin‐induced AKI and to determine the involved mechanisms.

## 2 MATERIALS AND METHODS

### 2.1 Animals

The study was approved by Animal Care and Use Ethics Committee of Sichuan University. FABP4 knockout mice (C57BL/6J background) were purchased from Model Animal Research Center of Nanjing University (Nanjing, China). Target site was in exon 2 of FABP4 gene, and target sequences were used (Figure S1). Male C57BL/6J mice, FABP4 WT and FABP4 KO mice were housed in a controlled environment (constant temperature at 20 ± 20°C and humidity at 50%‐60% with a 12‐hour light and 12‐hour dark cycle) and were free access to food and water. The mice were housed for 1 week of adaptation before further research. The animal experiments were taken place in the Animal Experiment Center of West China Hospital.

### 2.2 Primary antibodies

Primary antibodies were as follows: anti‐FABP4 (JM10‐99; HuaBio), anti‐BAX (ab32503; Abcam), anti‐BCL‐2 (ab3214; Abcam), anti‐BCL‐XL (ab32370; Abcam), anti‐cleaved caspase 3 (66470‐2‐Ig; Proteintech Group), anti‐p‐JNK (4668; Cell Signaling Technology), anti‐JNK (ab208035; Abcam), anti‐p‐eIF2α (ET1603‐14; HuaBio), anti‐eIF2α (RT1196; HuaBio), anti‐p‐PERK (sc32577; Santa Cruz), anti‐GRP78 (ER40402; HuaBio), anti‐IRE1α (ab48187; Abcam), anti‐CHOP (2895; Cell Signaling Technology), anti‐ATF4 (11815; Cell Signaling Technology), anti‐ATF6 (ER1706‐34; HuaBio), anti‐XBP1 (ab37152; Abcam) and anti‐caspase 12 (ab62484; Abcam).

### 2.3 Cisplatin treatment of mice

C57BL/6J mice (male, 8 weeks old) were intraperitoneally injected with 20 mg/kg body weight cisplatin, and control mice received an intraperitoneal injection of equal volume of 0.9% saline. As for FABP4 inhibitor (FABP4i) BMS309403 group, FABP4i was dissolved in PEG_400_, diluted in 0.9% saline and orally administered at a dose of 40 mg/kg/d for 3 days before the cisplatin injection. FABP4 WT and KO mice (male, 8 weeks old) were randomly divided into four groups: WT control or cisplatin groups, and KO control or cisplatin groups. The mice were also injected intraperitoneally with 20 mg/kg body weight cisplatin, and control mice received an intraperitoneal injection of the equal volume of 0.9% saline. Mice were killed at 72 hours after cisplatin injection, blood sample was collected for serum creatinine (sCr) and blood urea nitrogen (BUN) measurement, and the upper half of the left kidney was quickly removed and fixed in 10% phosphate‐buffered formalin for periodic acid‐Schiff (PAS) staining and TUNEL assay. The lower half of the left kidney was fixed in 2.5% glutaraldehyde for 2 hours at 4°C and processed for transmission electron microscope. The right kidney was quickly removed and frozen in liquid nitrogen and then stored at −80°C.

### 2.4 Serum analysis

The sCr and BUN levels were evaluated by automatic biochemical analyser (Mindray BS‐240). The AKI model was considered to be established when the level of sCr of the cisplatin‐treated group rose up to two times of their control littermates.

### 2.5 Histologic examination

The upper half of the left kidney, fixed in 10% phosphate‐buffered formalin, was dehydrated in a graded series of alcohol concentrations and embedded in paraffin. Kidney blocks were cut into 4‐μm sections and then subject to PAS staining for morphologic analysis. PAS stained tissue sections were viewed by light microscopy at magnifications of ×200 or ×400. For semiquantitative analysis of morphological changes, two sections were randomly selected from each sample of at least 3 for every group and 10 fields were randomly selected at a magnification of ×200 from each section in PAS staining. Histopathological changes were evaluated by the percentage of injured/damaged renal tubules, as indicated by tubular lysis, dilation, disruption and cast formation. Tissue injury was scored on a scale of 0‐4, with 0, 1, 2, 3 and 4 corresponding to 0%, <25%, 26%‐50%, 51%‐75% and >76% of injured/damaged renal tubules, respectively. Ten fields of ×40 magnification were examined and averaged.

### 2.6 Immunoblot analysis

Proteins were extracted from kidneys or HK‐2 cells using RIPA lysis buffer (P0013B; Beyotime Biotechnology) containing 4% cocktail proteinase inhibitors. After centrifugation at 3000 *g* for 15 minutes at 4°C, the supernatant was collected, and protein concentration was determined using Pierce^™^ BCA Protein Assay Kit (23225; Thermo Scientific). Bovine serum albumin was used as the standard. Equal amounts of protein lysate were loaded directly on 10%‐12% SDS‐PAGE and transferred onto PVDF membrane for protein blotting (162‐0177; Bio‐Rad). The membranes were blocked with 5% non‐fat dry milk (*w*/*v*) in TBS‐T for 1 hour at room temperature and then incubated with indicated primary antibodies overnight at 4°C. After being rinsed thrice with TBS‐T at 5‐minute intervals, the membranes were incubated with horseradish peroxidase‐labelled goat anti‐rabbit IgG (HA1001, 1:2000 dilution; HuaBio) or goat antimouse IgG (HA1006, 1:2000 dilution; HuaBio) for 1 hour. Immunoblots were visualized using the Immobilon Western Chemiluminescent HRP Substrate (WBKLS0500; Millipore Corporation) with Bio‐Rad ChemiDoc MP. All immunoblot analysis data are from experiments performed in triplicate. Densitometry analysis was performed using ImageJ 6.0 software (National Institutes of Health).

### 2.7 Immunofluorescence staining

Renal specimens were embedded in OCT compound, frozen in acetone‐dry ice mixture and cut into 3‐ to 5‐μm section on a cryostat and stored at −80°C until use. Non‐specific binding sites were blocked with PBS containing 5% bovine serum for 1 hour at room temperature. For staining, we incubated the specimens overnight with the first primary antibody at 4°C. After washing with PBS, the corresponding secondary antibody was applied for 1 hour. The samples were washed with PBS, stained with DAPI (D8200; Solarbio) and mounted with cover clips. In negative controls, primary antibodies were replaced by PBS. Secondary antibodies (1:500 dilution; Jackson ImmunoResearch) matched with a corresponding primary antibody were used to display fluorescent signals. Images were exported from ZEN 2012 microscopy software (blue edition).

### 2.8 Electron microscopy

After being fixed in cold 2.5% glutaraldehyde for 2 hours at 4°C, kidney tissues were washed with phosphate‐buffered saline (PBS; 0.2 mol/L, pH 7.4) for 2 hours, fixed with 1% osmic acid for 2 hours and then washed six times with PBS for 10 minutes per wash. The samples were dehydrated with ethanol and cleaned with epoxypropane. They were embedded in EPON 812 overnight at room temperature. Ultrathin sections (40‐60 nm) were cut (EM UC61rt; Leica) and stained with uranyl acetate/lead citrate. These sections were subsequently visualized using a transmission electron microscope (H‐7650; Hitachi).

### 2.9 Quantitative real‐time PCR analysis

Total RNA from kidney tissues was extracted using a total RNA extraction kit (TP‐01121; Foregene) according to the protocols. The concentration of mRNA was tested using a Scan Drop 100 (Analytik Jena) determiner. Quantitative real‐time PCR was performed after reverse transcription by using the fast qPCR kit (KK4610; Kapa Biosystems) in a PCR system (CFX Connect; Bio‐Rad). Target sequences were listed in Table S1. Relative expression levels were normalized to GAPDH.

### 2.10 TUNEL assay

In vivo, the terminal deoxynucleotidyl transferase‐mediated dUTP nick end labelling (TUNEL) staining was conducted on paraffin‐embedded slides using the DeadEnd^™^ Fluorometric TUNEL System (G3250; Promega) according to the experimental protocol. The sections were then incubated with DAPI (D8200; Solarbio) at a dilution of 1:500. Images were exported by fluorescence microscopy at magnifications of ×400. Positive cells were counted at magnification of ×200, and at least 10 fields per section for each sample were examined. In vitro, TUNEL assay was performed using the One Step TUNEL Apoptosis Assay Kit (C1086; Beyotime Biotechnology) according to the experimental protocol. The total number of TUNEL‐positive cells was calculated in ≥3 fields of view.

### 2.11 Cell culture and cisplatin treatment

Human renal proximal tubule cell line (HK‐2 cell) was a gift from Prof. Xueqing, Yu (The first Affiliated Hospital, Sun Yat‐sen University) and maintained in Dulbecco's modified Eagle's medium (DMEM)/F12 (SH30023.01B; Hyclone) supplemented with 10% foetal bovine serum (SH30084.03; Hyclone) at 37°C under humidified atmosphere of 5% CO_2_/95% air. We divided the cells in exponential growth state into five groups: cisplatin (20 μg/mL); cisplatin + FABP4i, incubated with FABP4i at 10 μmol/L 30 minutes prior to cisplatin treatment; cisplatin + sodium 4‐phenylbutyrate (4‐PBA, 11323; Cayman Chemical), incubated with 4‐PBA at 2 mmol/L 30 minutes prior to cisplatin treatment; cisplatin + tunicamycin (11445; Cayman Chemical), incubated with tunicamycin (Tm) at 25 ng/mL 30 minutes prior to cisplatin treatment; and control cells were incubated with complete medium alone.

### 2.12 Annexin V‐FITC/PI assay

The Annexin V‐FITC apoptosis analysis kit (AO2001‐02P‐H; SUNGENE) was used to assess apoptosis. HK‐2 cells were seeded into six‐well plates. The adhered cells were exposed to different media for 24 hours and then detached with trypsin. Subsequently, the cells were resuspended in a binding buffer and stained with Annexin V‐FITC (5 μL) and propidium iodide (PI, 5 μL) at room temperature for 15 minutes in the dark. Apoptotic cells were determined using a flow cytometer (Beckman Cytoflex; Beckman Coulter Australia Pty Ltd.). Annexin V‐FITC‐positive and PI‐negative cells were considered apoptosis.

## 3 RESULTS

### 3.1 FABP4i BMS309403 protected against cisplatin‐induced AKI

**Figure 1:**
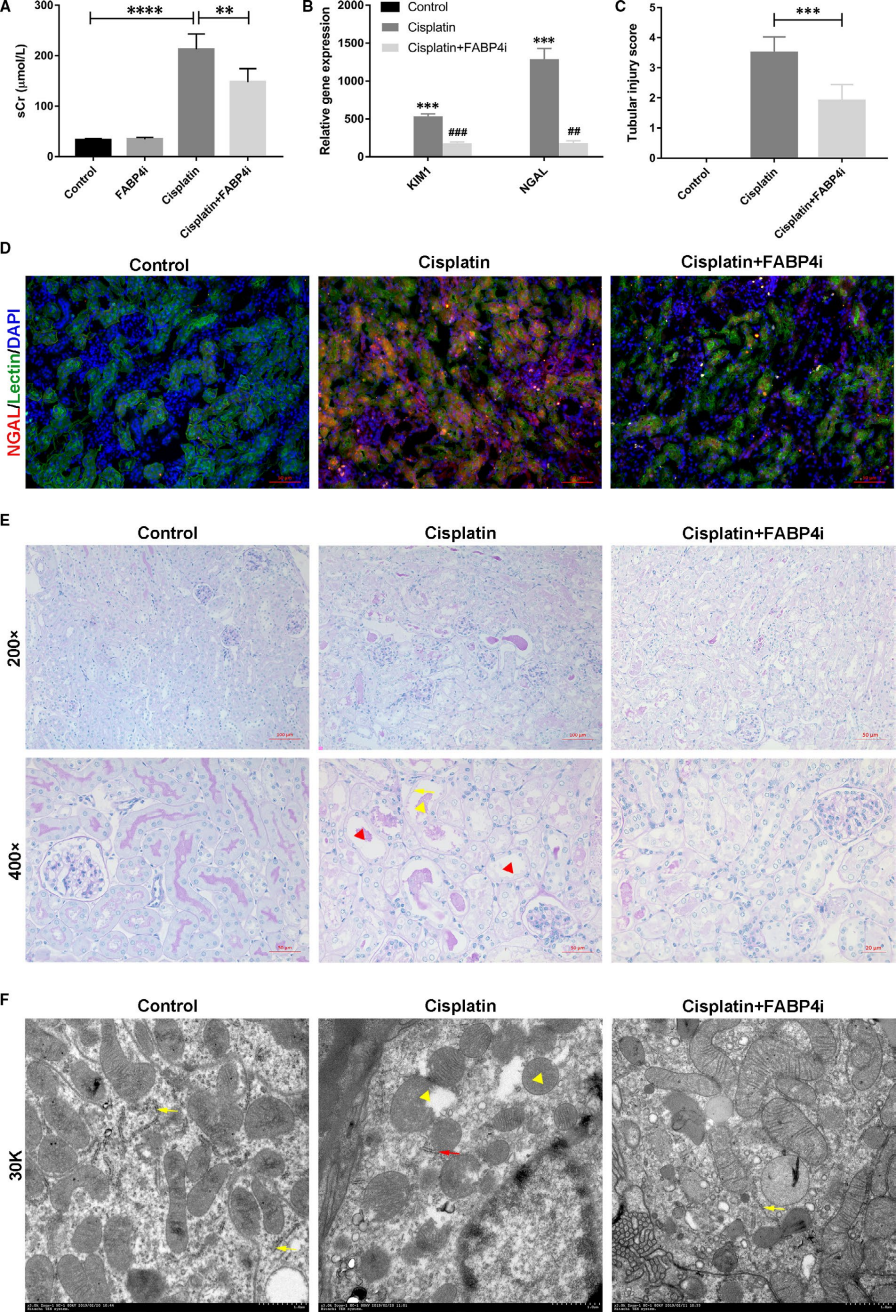
Treatment by a highly selective fatty acid‐binding protein 4 (FABP4) inhibitor BMS309403 alleviated cisplatin‐induced acute kidney injury (AKI). A, Serum creatinine (sCr). B, Relative mRNA expression of KIM1 and NGAL in kidney tissues. C, Tubular injury score evaluated by kidney periodic acid‐Schiff (PAS) staining. D, Immunofluorescence staining of NGAL and lectin in the kidney tissue. NGAL (red) was used as an AKI marker and lectin (green) as a marker of tubular epithelial cells. E, PAS staining of the kidney tissues (×200 and ×400). Red triangle: tubular dilatation; yellow triangle: cast formation; yellow arrow: loss of brush border. F, Photomicrographs were collected by transmission electron microscope (30 K). Yellow arrow: normal endoplasmic reticulum (ER); red arrow: expansion of ER; yellow triangle: mitochondrial swelling. Data are represented as the means ± SE (n = 6). **P < 0.01, ***P < 0.001, ****P < 0.0001 vs Control; ^##^ *P* < 0.01, ^###^ *P* < 0.001 vs Cisplatin

To confirm whether FABP4i BMS309403 possess renoprotective effect, we evaluated renal function and pathological changes in kidney tissues in a mouse model of cisplatin‐induced AKI. As exhibited in Figure 1, serum creatinine (sCr), renal mRNA levels of KIM1 and NGAL were markedly elevated at 3 days after cisplatin injection. In Figure 1B, the relative gene expressions of KIM1 are 1 in the control group, and those of KIM1 in Cisplatin and Cisplatin + FABP4 are 534.37 and 179.90, respectively. Similarly, the relative gene expressions of kidney injury marker NGAL are 1 in the control group, and those of NGAL in Cisplatin and Cisplatin + Fabp4 are 1287.62 and 184.78, respectively. Treatment of FABP4i at a dose of 40 mg/kg/d significantly improved acute renal dysfunction with good safety (Figure S2). Immunofluorescence staining of renal injury maker NGAL in the tubular epithelial cells (lectin) of kidney tissues confirmed that oral administration of FABP4i alleviated cisplatin‐induced AKI (Figure 1D). Consistently, the result of PAS‐stained kidneys showed less tubular dilatation, swelling, cast formation and preservation of a brush border in the FABP4i‐treated mice as compared to that of cisplatin‐induced group (Figure 1C,E). By transmission electron microscopy, we observed the mitochondrial swelling and loss of brush border in cisplatin group, and FABP4i administration improved mitochondrial swelling and injury (Figure 1F).

**Figure 2:**
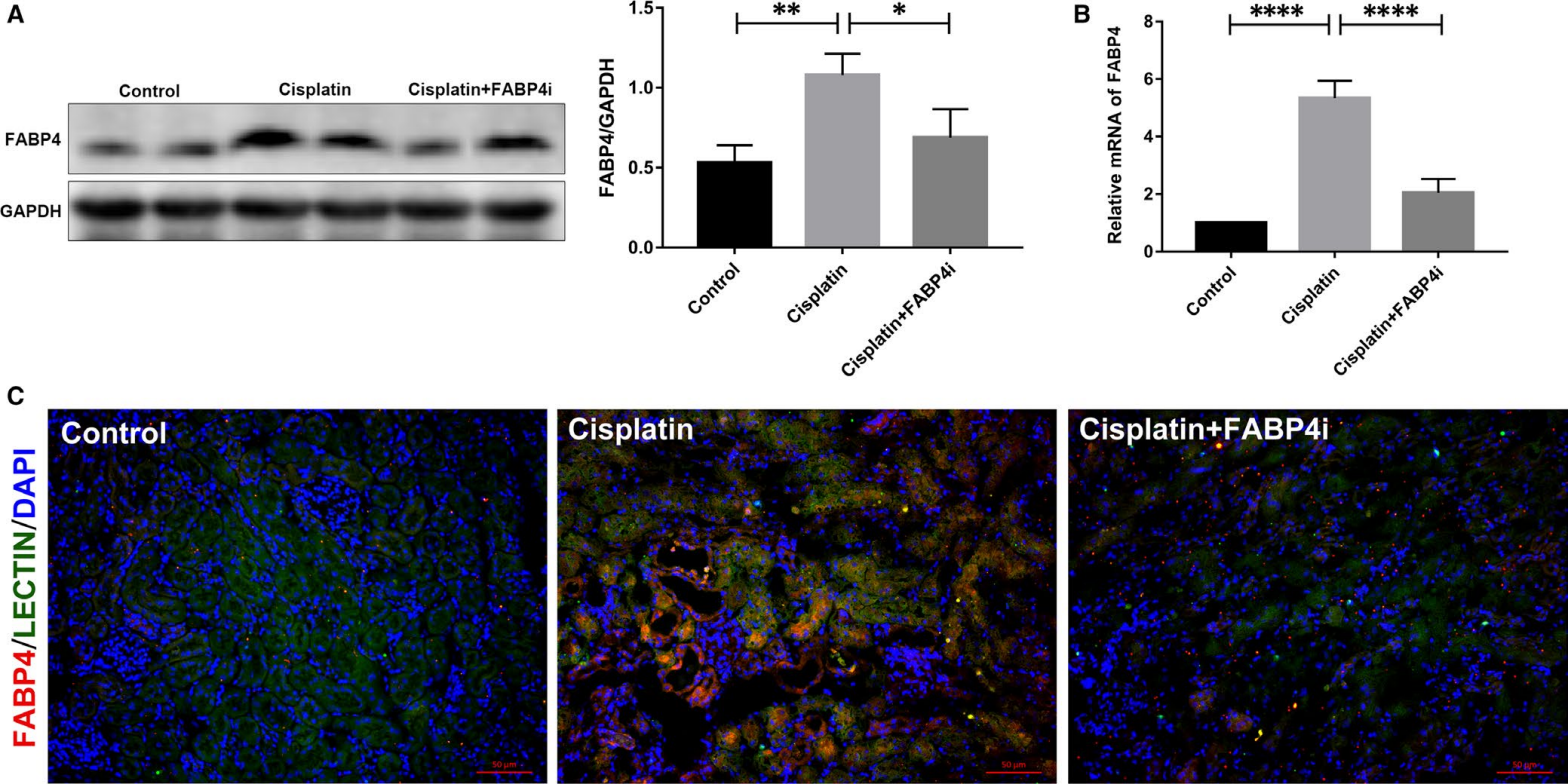
The expression of fatty acid‐binding protein 4 (FABP4) in the kidney tissue of cisplatin‐induced acute kidney injury (AKI). A, The kidney tissue lysates were subjected to immunoblot analysis with indicated antibodies against FABP4. B, Relative mRNA expression of FABP4 in kidney tissues. Expressions of FABP4 was quantified by densitometry and normalized with GAPDH. C, Immunofluorescence staining of FABP4 (red) in the tubular epithelial cells (lectin, green) of cisplatin‐induced AKI. Data are represented as the means ± SE (n = 3). *P < 0.05, **P < 0.01, ****P < 0.0001

To determine whether BMS309403 exhibited renoprotective effect by targeting FABP4, we further evaluated the FABP4 activity in the kidney tissues of cisplatin‐induced AKI. As shown in Figure 2, renal FABP4 protein and mRNA expression were markedly elevated at 3 days after cisplatin injection, and treatment of FABP4i at a dose of 40 mg/kg/d for 3 days significantly inhibited FABP4 protein expression and relative mRNA level in the injured kidney tissue.

Increasing evidence showed that tubular epithelial cells played multiple roles in renal repair or progression to AKI and chronic kidney disease (CKD).^25^ To further investigate whether FABP4 was expressed in renal tubular epithelial cells, renal tissues were stained by FABP4 and a proximal epithelial cell marker lectin. As exhibited in Figure 2C, FABP4 was rarely expressed in the control group, but notably overexpressed in the cisplatin‐injected group and mainly merged with lectin. Oral administration of FABP4i significantly inhibited the FABP4 protein expression, which was consistent with the result of immunoblot analysis. So, these findings suggested that cisplatin induced FABP4 overexpression in tubular epithelial cells, and BMS309403 suppressed the FABP4 activity.

### 3.2 Inhibition of FABP4 activity reduced apoptosis in the kidneys of cisplatin‐injured AKI

**Figure 3:**
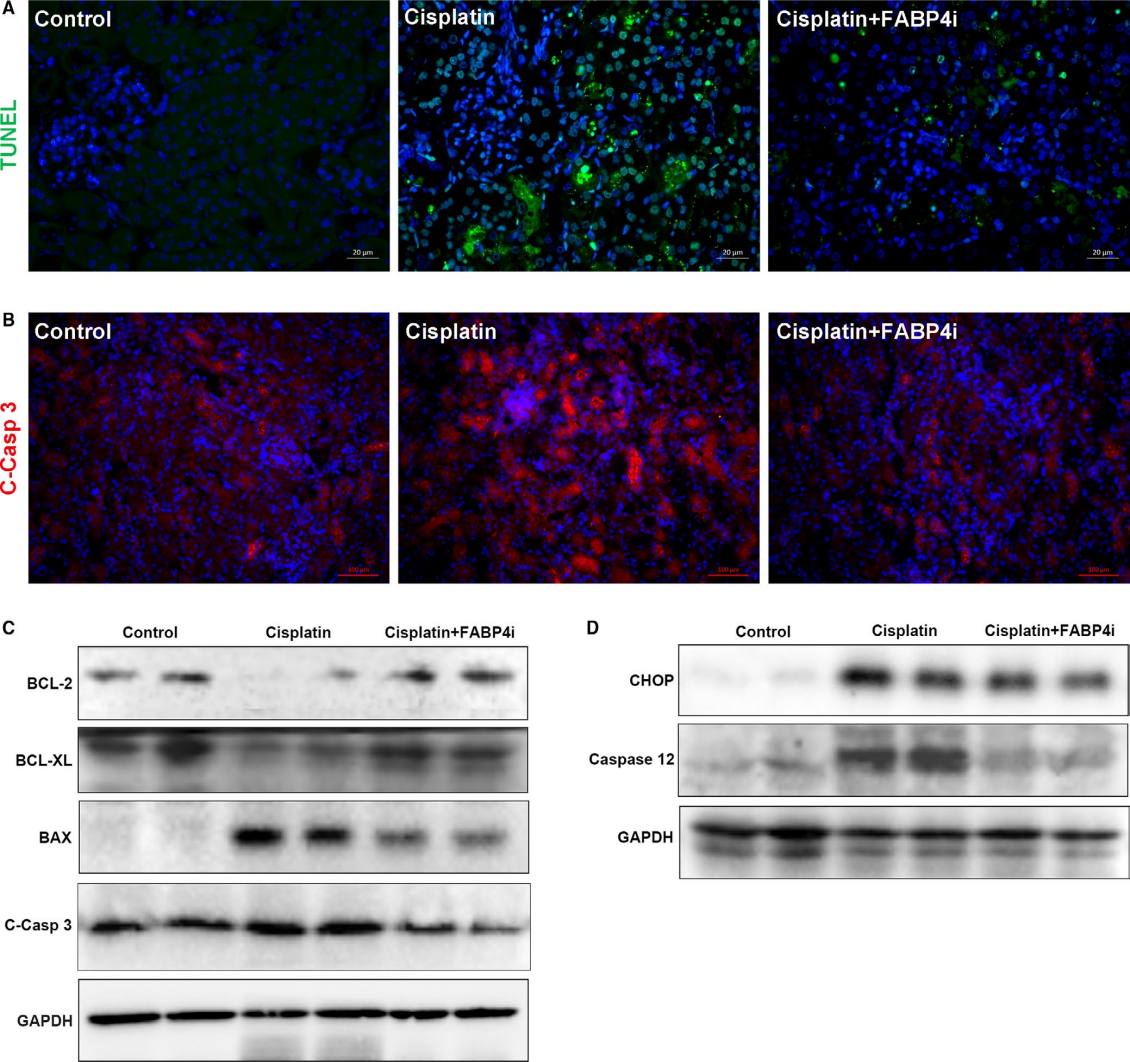
Inhibition of fatty acid‐binding protein 4 (FABP4) activity attenuated cisplatin‐induced apoptosis in the kidney of acute kidney injury. A, TUNEL staining of kidney tissues (green: TUNEL‐positive cells, ×400). B, Immunofluorescence staining was performed to detect the expression of cleaved caspase 3 in kidney tissue sections (red, ×400). C, The kidney tissue lysates were subjected to immunoblot analysis with antibodies against BCL‐2, BCL‐XL, BAX and cleaved caspase 3 (C‐Casp 3). D, The kidney tissue lysates were subjected to immunoblot analysis with antibodies against CHOP and Caspase 12

Renal tubular cell apoptosis was a prominent feature in cisplatin‐induced AKI.^26^ To investigate the role of BMS309403 in the model, we analysed the apoptosis by TUNEL staining. As expected in Figure 3A, the large number of TUNEL‐positive cells was observed in the cisplatin‐injured kidney and FABP4i administration diminished the number of TUNEL‐positive cells.

Caspase 3 was the key executioner which modified proteins responsible for apoptosis. Activated caspase 12 cleaved procaspase 9 to activate procaspase 3.^5^ In Figure 3B, the results of immunofluorescence staining showed that cisplatin induced the high expression of cleaved caspase 3 in kidney tissues and FABP4i treatment significantly reduced its level. Consistent with our above‐mentioned results, the injection of cisplatin induced cell apoptosis, as assessed by the up‐regulation of BAX and cleaved caspase 3, and the down‐regulation of BCL‐2 and BCL‐XL. However, FABP4i administration provided a marked renal protection against cisplatin‐induced apoptosis, by restoring the expression of apoptosis‐related signal markers (Figure 3C). The quantification of apoptosis‐related protein levels was shown in Figure S3.

Several components of caspase cascade were involved in ER stress‐induced apoptosis. Especially, caspase 12, which was related to the ER membrane, was a proximal regulator for ER stress‐induced caspase activation followed by apoptosis. ER stress also could activate caspase 12‐dependent CHOP apoptotic pathway. In Figure 3D, we found that the caspase 12 and CHOP protein were highly expressed in the cisplatin‐induced AKI. Similarly, FABP4i treatment down‐regulated the two protein levels to reduce apoptosis.

### 3.3 Pharmacological inhibition of FABP4 activity suppressed ER stress in cisplatin‐induced AKI

**Figure 4:**
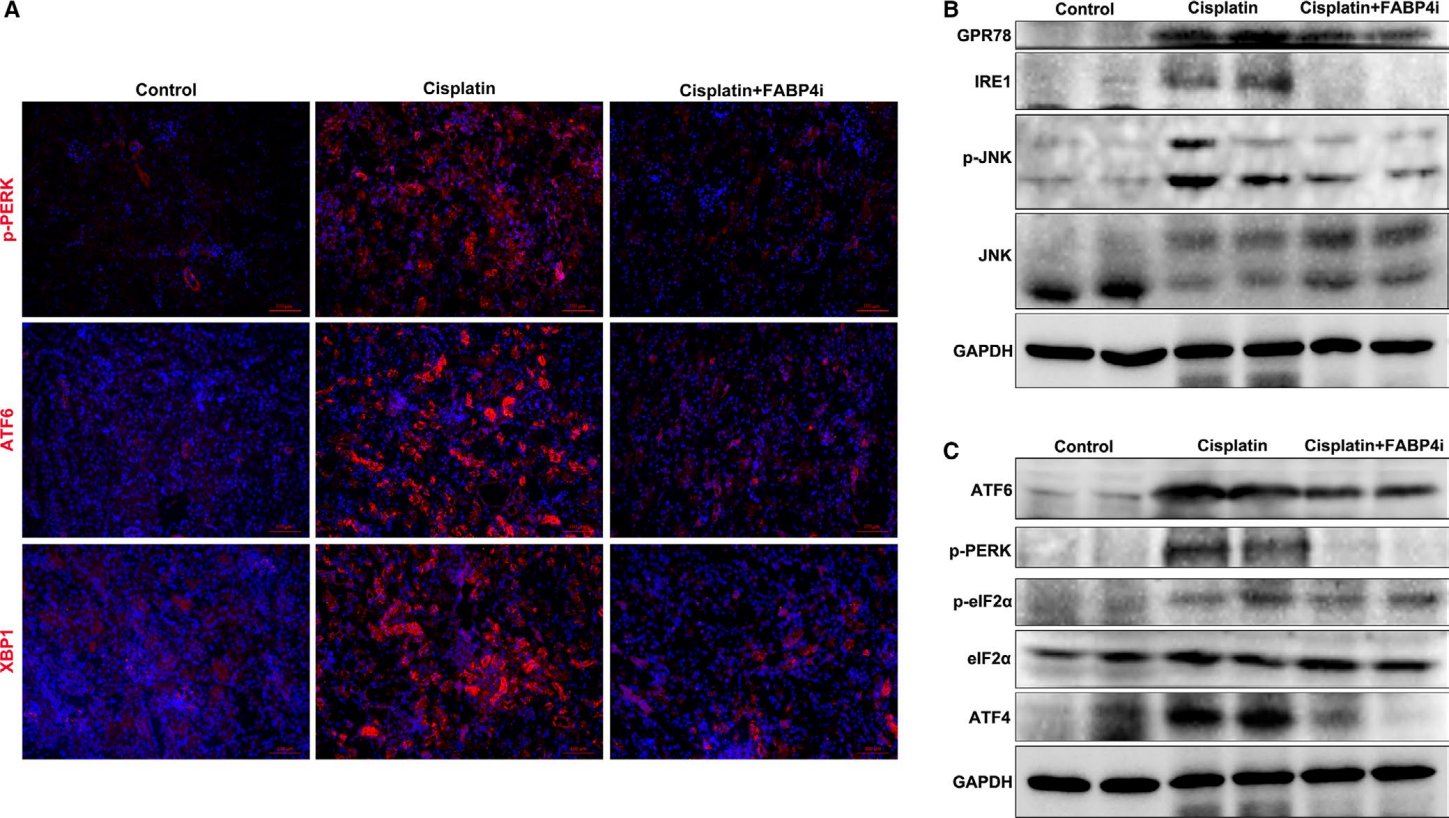
Pharmacological inhibition of fatty acid‐binding protein 4 (FABP4) attenuated renal endoplasmic reticulum stress. A, Immunofluorescence staining of ER stress‐related p‐PERK, ATF4 and XBP1 treated by FABP4 inhibitor (FABP4i) in the kidneys of cisplatin‐induced acute kidney injury. B, The protein expression of GRP78, IRE1 and p‐JNK/JNK as measured by immunoblot analysis, in kidney tissue sections in each group. C, The protein expression of ATF6, p‐PERK, p‐eIF2α/eIF2α and ATF4 as measured by immunoblot analysis, in kidney tissue sections in each group

Perturbations of kidney cells in AKI resulted in the accumulation of unfolded and misfolded proteins in the ER, leading to UPR or ER stress.^27^ Firstly, we evaluated ER stress in kidney tissues by the expression of three UPR‐related proteins, XBP1, p‐PERK and ATF4, which were highly expressed in cisplatin‐injured kidney tissues by immunofluorescence staining. After FABP4i treatment, the corresponding expression of three proteins was effectively suppressed (Figure 4A).

To validate the hypothesis that FABP4i alleviated cisplatin‐induced AKI by the suppression of ER stress, we furtherly conducted immunoblot analysis of ER stress‐related three pathways of IRE1, PERK and ATF6, as well as JNK. In Figure 4B, we found that the expression of IRE1‐XBP1 was markedly elevated in kidney at 3 days after cisplatin injection. Treatment of FABP4i at a dose of 40 mg/kg/d for 3 days significantly improved ER tress. As shown in Figure 4C, the phosphorylation PERK and eIF2α as well as the downstream ATF4 were also highly expressed in the cisplatin‐injured kidney tissues. After FABP4i treatment, the key protein expression of PERK pathway was effectively suppressed. The quantification of ER stress‐related protein levels was shown in Figure S4. Taken together, these findings suggested that cisplatin induced renal ER stress and FABP4i administration inhibited renal ER stress activation in cisplatin‐induced AKI.

The IRE1 complex activated JNK pathway to play a role in inflammation.^28^, ^29^ The JNK phosphorylation (Figure 4B) with pro‐inflammatory cytokine mRNA level of IL‐1β and IL‐6 (Figure S5) was also induced in the cisplatin‐injured kidneys. Meanwhile, FABP4i reduced cisplatin‐induced p‐JNK expression and alleviated renal inflammation.

### 3.4 Genetic inhibition of FABP4 suppressed ER stress and apoptosis in kidneys of cisplatin‐induced AKI

**Figure 5:**
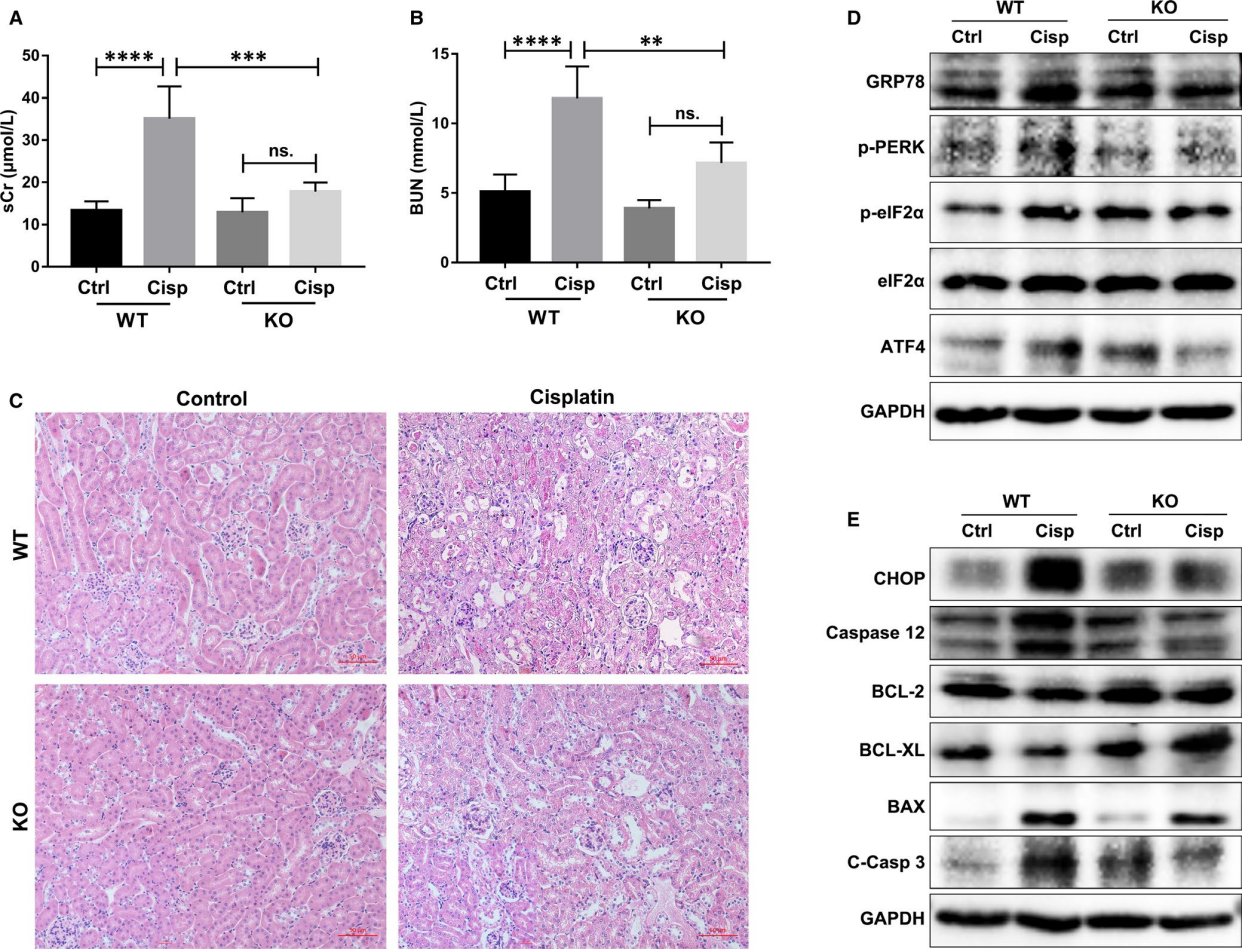
Genetic inhibition of fatty acid‐binding protein 4 (FABP4) attenuated cisplatin‐induced acute kidney injury by the regulation of endoplasmic reticulum stress‐mediated apoptosis. A, B, The levels of sCr and blood urea nitrogen (BUN). C, H&E staining of the kidney tissues (200× and 400× displayed in left bottom). D, The protein expression of GRP78, p‐PERK, p‐eIF2α/eIF2α and ATF4 as measured by immunoblot analysis, in kidney tissues in each group. E, The protein expression of CHOP, caspase 12, BCL‐2, BCL‐XL, BAX and cleaved caspase 3 (C‐Casp 3) as measured by immunoblot analysis, in kidney tissues in each group. Ctrl, control; Cisp, Cisplatin. Data are represented as the means ± SE (n = 6). **P < 0.01, ***P < 0.001, ****P < 0.0001

To determine the functional relevance of cisplatin responsive FABP4 induction, we examined the inhibitory effect of FABP4 gene deficiency against cisplatin‐induced AKI. As shown in Figure 5A,B, wild‐type FABP4 mice with cisplatin injection developed renal dysfunction as indicated by the increased levels of sCr and BUN throughout the experimental period. Consistent with the findings of FABP4i, genetic inhibition of FABP4 also protected against cisplatin‐induced AKI. No significant differences in sCr and BUN levels were found between FABP4 KO and cisplatin‐induced FABP KO mice. Cisplatin injection alone caused severe tubular dilatation, swelling, necrosis, cast formation and preservation of a brush border in WT mice; however, the disruption on the kidney tissues of FABP4 KO mice was mild (Figure 5C).

Similar to cisplatin‐induced C57BL/6J mice, cisplatin also triggered the responsive expression of UPR molecule (GRP78 and CHOP), ER stress PERK‐eIF2α‐ATF4 pathway and apoptosis‐related proteins in the injured kidneys of WT FABP4 mice. Notably, genetic knockout of FABP4 attenuated ER stress in cisplatin‐induced elevation of GRP78, CHOP, and the phosphorylation of PERK and eIF2α, as well as ATF4 protein expression (Figure 5D). The cisplatin responsive induction of apoptosis‐related proteins in kidneys, including caspase 12, BCL‐2, BCL‐XL, BAX and cleaved caspase 3, was also dramatically regulated in FABP4 KO mice (Figure 5E). The quantification of ER stress and apoptosis‐related protein levels was shown in Figure S6. These results suggested that FABP4 deficiency could attenuate cisplatin‐induced AKI by regulation of ER stress and apoptosis.

### 3.5 BMS309403 inhibited FABP4 activity to improve ER stress‐induced apoptosis in cisplatin‐stimulated HK‐2 cells

**Figure 6:**
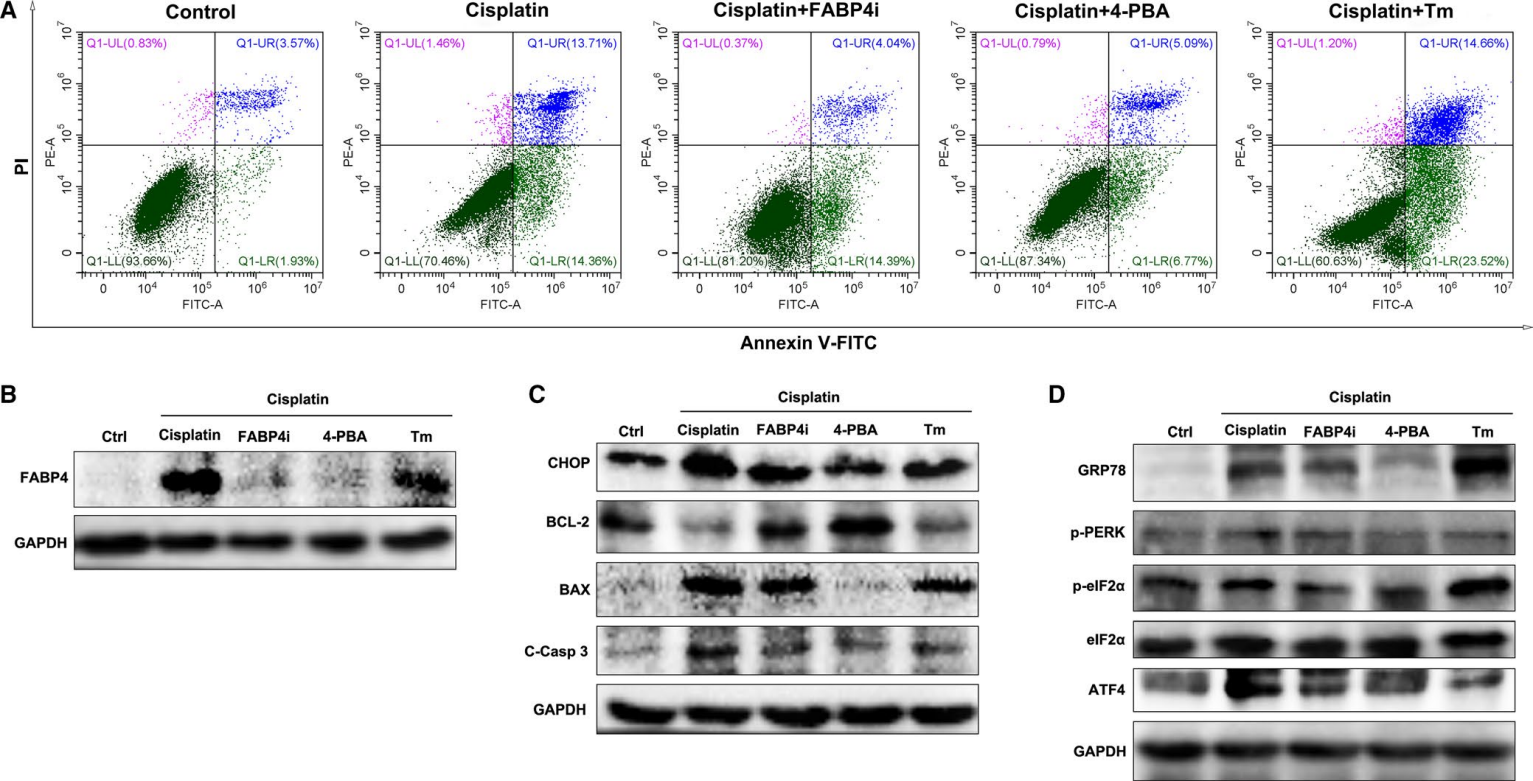
Inhibition of fatty acid‐binding protein 4 (FABP4) activity suppressed endoplasmic reticulum stress‐mediated apoptosis in cisplatin‐induced renal proximal tubule HK‐2 cells. A, HK‐2 cells were treated with FABP4i, subjected to Annexin V/PI staining and then analysed the apoptosis by flow cytometry. B, The protein expression of FABP4 in HK‐2 cells as measured by immunoblot analysis. C, The protein expression of CHOP, BCL‐2, BAX and cleaved caspase 3 in HK‐2 cells. D, The protein expression of GRP78, p‐PERK, p‐eIF2α/eIF2α and ATF4 in HK‐2 cells as measured by immunoblot analysis

Furthermore, we explored whether FABP4i in vitro suppressed ER stress and apoptosis in cisplatin‐stimulated human proximal tubule epithelial HK‐2 cells. 4‐PBA is a chemical chaperon to attenuate ER stress, and tunicamycin is a naturally occurring antibiotic to induce ER stress in cells. After cisplatin stimulation, HK‐2 cells showed a significant cell apoptosis and FABP4i inhibited the number of apoptotic cells (Figure 6A and Figure S8). Meanwhile, ER stress inhibitor 4‐PBA also reduced the number of apoptotic cells and ER stress inducer tunicamycin aggravated the apoptosis as compared to that of cisplatin.

As shown in Figure 6B, the results indicated that FABP4i in vitro suppressed the cisplatin‐induced FABP4 protein expression. In the cisplatin‐stimulated HK‐2 cells, the protein expressions of two UPR molecules CHOP, GRP78 and the PERK‐eIF2‐ATF4 pathway as well as apoptosis‐related proteins were induced. FABP4i treatment accordingly inhibited the key mediator levels of ER stress‐induced apoptosis, which were consistent with that of 4‐PBA. However, ER stress inducer tunicamycin exhibited the opposite effects against FABP4i and 4‐PBA by immunoblot analysis (Figure 6C,D). The quantification of ER stress‐related protein levels was shown in Figure S7.

## 4 DISCUSSION

For several forms of AKI, therapeutics for kidney injury are not effective due to the unpredictable and intrinsic disease nature.^6^ On the other hand, cisplatin is a known chemotherapeutic agent with nephrotoxicity whose administration is scheduled, and thus, renal protective strategy is feasible. In our previous study, a highly selective FABP4i BMS309403 has reported to alleviate renal tubular damage against ischaemia/reperfusion‐ and rhabdomyolysis‐induced AKI. FABP4 has been confirmed to contribute to ischaemia/reperfusion‐ and rhabdomyolysis‐induced AKI, and FABP4 inhibition might be a potential strategy against AKI. In the study, pharmacological and genetic inhibition of FABP4 treatment significantly attenuated kidney function and renal tubular damage by the regulation of ER stress‐mediated apoptosis in the kidneys of cisplatin‐induced AKI.

Apoptosis is usually a response to cell microenvironment.^30^ Apoptosis requires the lethal molecule activation and the inactivation of pro‐survival ones. Apoptotic pathways in the tubular epithelium could be induced by caspase cascade activation, mitochondrial injury and ER stress.^31^ Apoptosis also promoted the loss of renal epithelial cells that characterized AKI.^5^ Accumulating evidence demonstrated that the activation of caspase 3 was predominant which responsible for renal tubular cell apoptosis in cisplatin‐induced AKI. In our study, selective blockage of FABP4 activity notably reduced the number of TUNEL‐positive cells, inhibited the cleavage of caspase 3 and up‐regulated BCL‐2, BCL‐XL level in the cisplatin‐injured kidneys and cisplatin‐stimulated HK‐2 cells. Therefore, FABP4 inhibition could reduce tubular epithelial cell apoptosis in the pathogenesis of cisplatin‐induced AKI.

ER stress is a cellular stress response to the unfolded or misfolded protein accumulation in the ER lumen, which is also called the UPR. Multiple stimulatory signals or conditions could trigger ER stress in AKI, including hypoxia, mutant protein aggregation, energy deprivation and metabolic dysfunction.^7^, ^27^, ^32^, ^33^, ^34^ Overwhelming ER stress induced apoptosis via three typical pathways, PERK, ATF6 and IRE1. Under UPR, PERK was released from its chaperone GRP78 to permit eIF2α phosphorylation, leading to the further activation of ATF4 and CHOP. Active IRE1 targeted the downstream XBP1 and JNK which resulted in apoptosis. In addition, caspase 12, which was only expressed in rodents, was another key molecule that responded to ER stress and induced the cleavage of caspase 3 to initiate apoptosis.^35^

Considerable evidence indicated a link between FABP4 and ER stress‐mediated apoptosis in diabetes and atherosclerosis.^18^, ^19^, ^36^, ^37^ However, the role of FABP4 in kidneys of cisplatin‐induced AKI is poorly understood. In our study, FABP4 deficiency markedly suppressed ER stress, as evidenced by the decreased protein expression of GRP78. The UPR was inhibited by FABP4i as indicated by the reduced expression of p‐PERK/p‐eIF2α/ATF4, ATF6 and IRE1 proteins. Moreover, FABP4i treatment down‐regulated the mediator expressions of ER‐induced apoptosis, including CHOP, p‐JNK and caspase 12. Gene knockout of FABP4 also regulated the ER stress p‐PERK/p‐eIF2α/ATF4 pathway and apoptosis‐related protein expressions. The consistent results were found in the in vitro study of cisplatin‐stimulated HK‐2 cells. Taken together, selective blockage of FABP4 by BMS309403 and genetic knockout of fabp4 exerted renoprotective effect against apoptosis via the inhibition of ER stress.

In our previous study, we reported for the first time that I/R surgery induced overexpression of FABP4 in tubular cells. Chemical inhibition of FABP4 by BMS309403 ameliorated renal structural damage and improved kidney function in I/R‐induced AKI. Moreover, we demonstrated that the inhibition of FABP4 attenuated tubular cells apoptosis and ER stress induced by renal I/R. Similarly, we also reported that BMS309403 pre‐treatment at a dose of 30 mg/kg/d for four consecutive days remarkably reduced serum creatinine, pro‐inflammatory cytokine expression of TNF‐α, IL‐6 and MCP‐1 as well as attenuated tubular damage in glycerol‐injured kidneys. Oral administration of BMS309403 attenuated renal ER stress in AKI and the related ERS protein expression of GRP78, CHOP, p‐PERK and ATF4.^38^ In contrast to cisplatin‐induced AKI, pharmacological and genetic inhibition of FABP4 also alleviated AKI by the modulation of apoptosis via the inactivation of ER stress in the tubular epithelial cells. Mechanically, the similarity of FABP4 in the three mice models of AKI could regulate ER stress, apoptosis and inflammation to improve acute kidney function.

In summary, the role of FABP4 in cisplatin‐induced kidney injury is a novel finding. The most significant thing in the study was that we confirmed pharmacological and genetic inhibition of FABP4 activity effectively alleviated cisplatin‐induced AKI. These findings also indicated that FABP4 inhibition reduced renal tubular cell apoptosis via the inactivation of ER stress in cisplatin‐induced AKI.

## CONFLICT OF INTEREST

No conflicts of interest, financial or otherwise, are declared by the authors.

## AUTHOR CONTRIBUTION

L.M. and P.F. participated in experimental design; Z.T., F.G., Z.H., Z.X., J.L., S.T., L‐Z.L. and X.D. conducted experiments; Z.T. and F.G. contributed new reagents or analytic tools; Z.T. and F.G. performed data analysis; Z.T., Y‐Y.F. and L.M. contributed to the writing of the manuscript.

## Data Availability

The data that support the findings of this study are available from the corresponding author upon reasonable request.
