# Supplementary material for: Pharmacological and genetic inhibition of fatty acid‐binding protein 4 alleviated cisplatin‐induced acute kidney injury
Source: J Cell Mol Med. 2019 Jul 8;23(9):6260–70. doi: 10.1111/jcmm.14512 (PMC6714212; doi:10.1111/jcmm.14512)
Supplement: Supplementary file 1 [file JCMM-23-6260-s001.pdf]

A

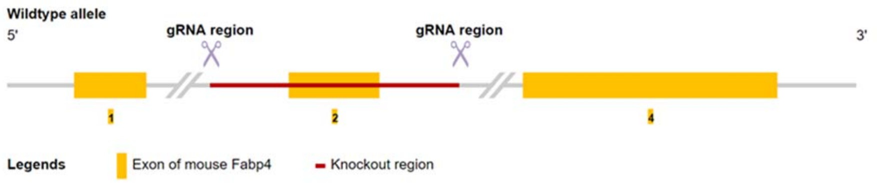

B

FABP4-5in-F1	GTACAGAATGCTGTGCTGTG	Wt:1725bp
FABP4-3in-R1	GGAACCTGGGTCCAAAGGAC	Ko:<1725bp
FABP4-5out-F2	GAAGACACAAGTGAAGCTGT	Wt:1770bp
FABP4-3in-R2	CAGGACAAGAACATAATCCA	Ko:<1770bp
FABP4-WT-F1	GACAGAAGGATTCTGTGTAACATGC	WT:410BP
FABP4-WT-R1	GGCCTCACTTAGAGTTATTGTGTTA	
FABP4-WT-F2	GAACAAGGAGCTGATGCAGAGTG	WT:239bp
FABP4-WT-R2	ACTCCACATGCACTACTGTATGAC	

C

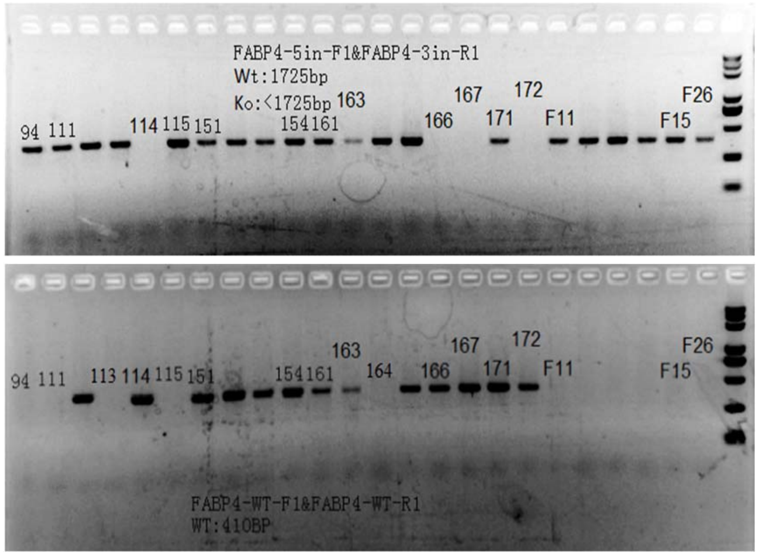

FABP4 KO/KO: 94, 111, 113, 115,164, F11-F15, F26;
FABP4 WT/WT:114, 166, 167, 172;
FABP4 KO/WT: the others.

Figure S1. FABP4 knockout mice. The design (A), target sequences (B) and identification (C) of FABP4 knockout mice.
