# Supplementary material for: Pharmacological and genetic inhibition of fatty acid‐binding protein 4 alleviated cisplatin‐induced acute kidney injury
Source: J Cell Mol Med. 2019 Jul 8;23(9):6260–70. doi: 10.1111/jcmm.14512 (PMC6714212; doi:10.1111/jcmm.14512)
Supplement: Supplementary file 2 [file JCMM-23-6260-s002.pdf]

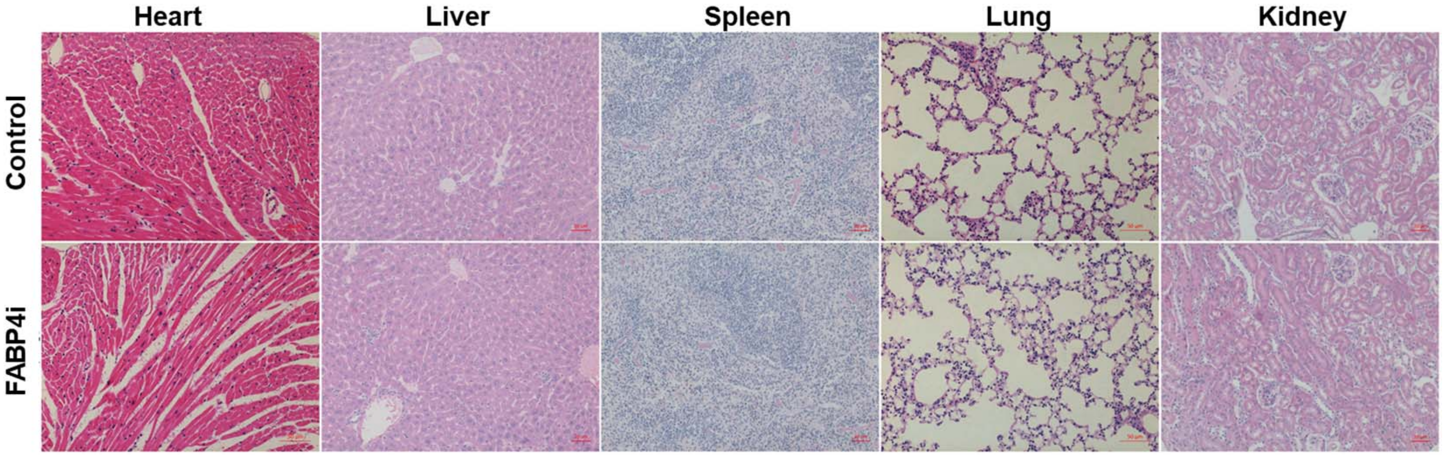

Figure S2. No pathologic changes of FABP4i BMS309403 on Heart, Liver, Spleen, Lung and Kidney tissues. FABP4i was orally administrated to C57BL/6J mice at a dose of 40 mg/kg/d for 3 day.
