# Supplementary material for: Pharmacological and genetic inhibition of fatty acid‐binding protein 4 alleviated cisplatin‐induced acute kidney injury
Source: J Cell Mol Med. 2019 Jul 8;23(9):6260–70. doi: 10.1111/jcmm.14512 (PMC6714212; doi:10.1111/jcmm.14512)
Supplement: Supplementary file 4 [file JCMM-23-6260-s004.pdf]

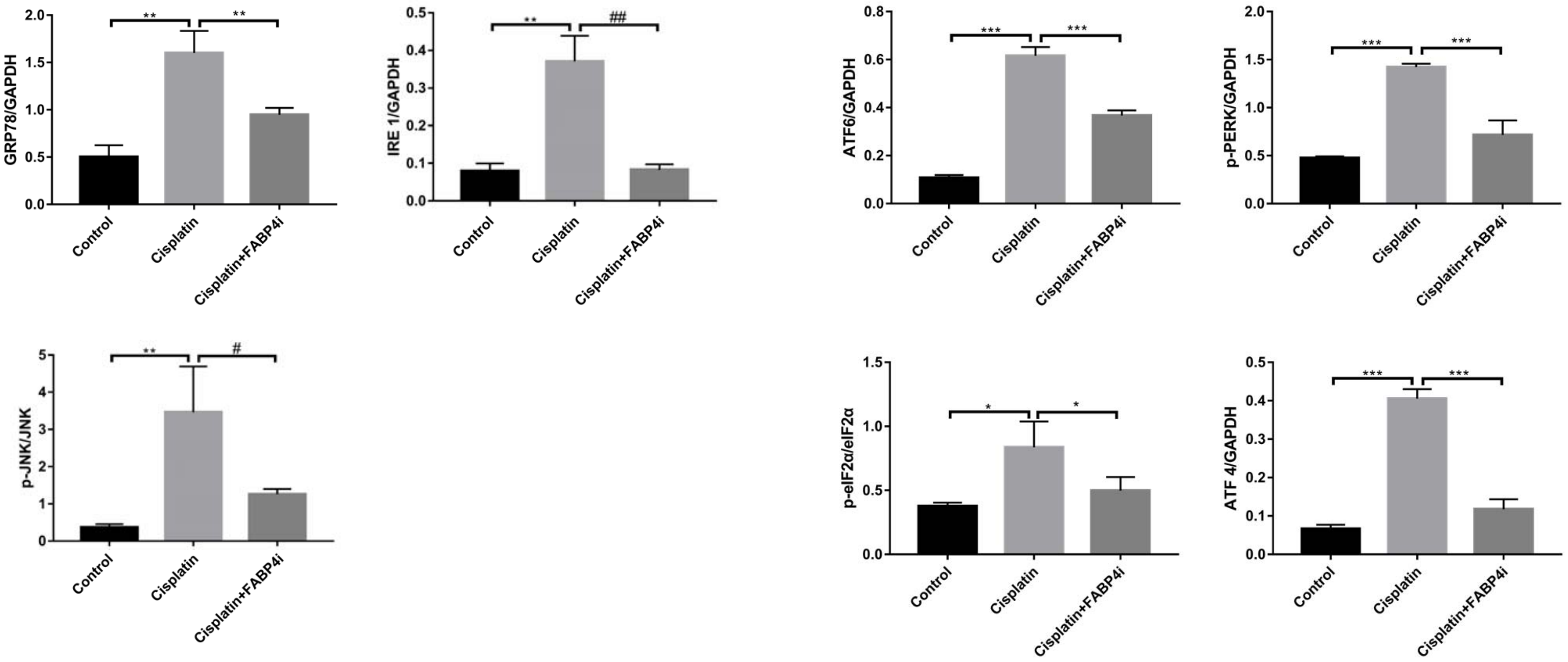

Figure S4. Densitometry values of ER stress-related proteins in FABP4i-treated AKI mice. The kidneys were taken for immunoblot analysis as shown in Figure 4. The densitometry values of proteins were normalized with GAPDH. Data expressed as means ± SD for groups of 3 independent experiments. *** $P < 0.001$; ** or ##$P < 0.01$; * or #$P < 0.05$.
