# Supplementary material for: Pharmacological and genetic inhibition of fatty acid‐binding protein 4 alleviated cisplatin‐induced acute kidney injury
Source: J Cell Mol Med. 2019 Jul 8;23(9):6260–70. doi: 10.1111/jcmm.14512 (PMC6714212; doi:10.1111/jcmm.14512)
Supplement: Supplementary file 5 [file JCMM-23-6260-s005.pdf]

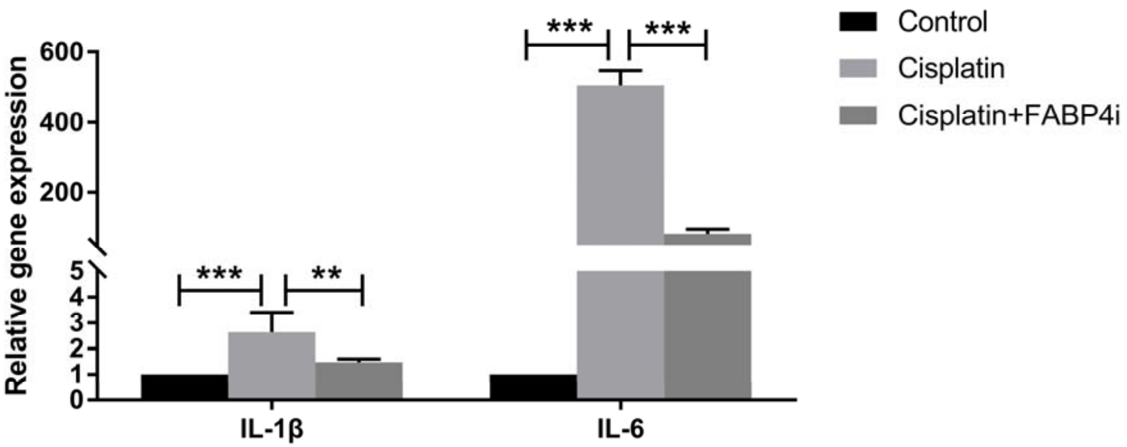

Figure S5. The mRNA expression of IL-1β and IL-6 in the kidneys of cisplatin-induced AKI. FABP4i was orally administrated to C57BL/6J mice at a dose of 40 mg/kg/d for 3 day. All data are represented as the means±SE (n=6). **P<0.01, ***P<0.001.
