# Supplementary material for: Pharmacological and genetic inhibition of fatty acid‐binding protein 4 alleviated cisplatin‐induced acute kidney injury
Source: J Cell Mol Med. 2019 Jul 8;23(9):6260–70. doi: 10.1111/jcmm.14512 (PMC6714212; doi:10.1111/jcmm.14512)
Supplement: Supplementary file 6 [file JCMM-23-6260-s006.pdf]

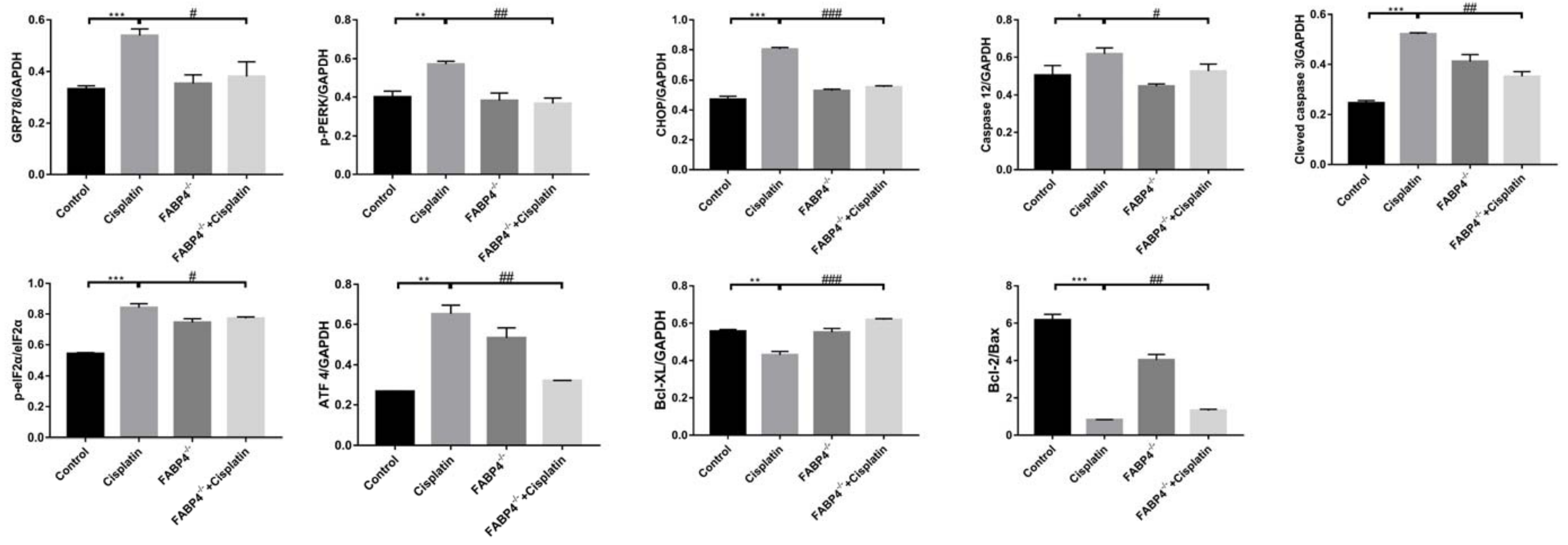

Figure S6. Densitometry values of ER stress-related proteins in FABP4 KO AKI mice. The kidneys were taken for immunoblot analysis as shown in Figure 5. The densitometry values of proteins were normalized with GAPDH. Data expressed as means ± SD for groups of 3 independent experiments. *** or ### P < 0.001; ** or ##P < 0.01; * or #P < 0.05.
