# Supplementary material for: Pharmacological and genetic inhibition of fatty acid‐binding protein 4 alleviated cisplatin‐induced acute kidney injury
Source: J Cell Mol Med. 2019 Jul 8;23(9):6260–70. doi: 10.1111/jcmm.14512 (PMC6714212; doi:10.1111/jcmm.14512)
Supplement: Supplementary file 8 [file JCMM-23-6260-s008.pdf]

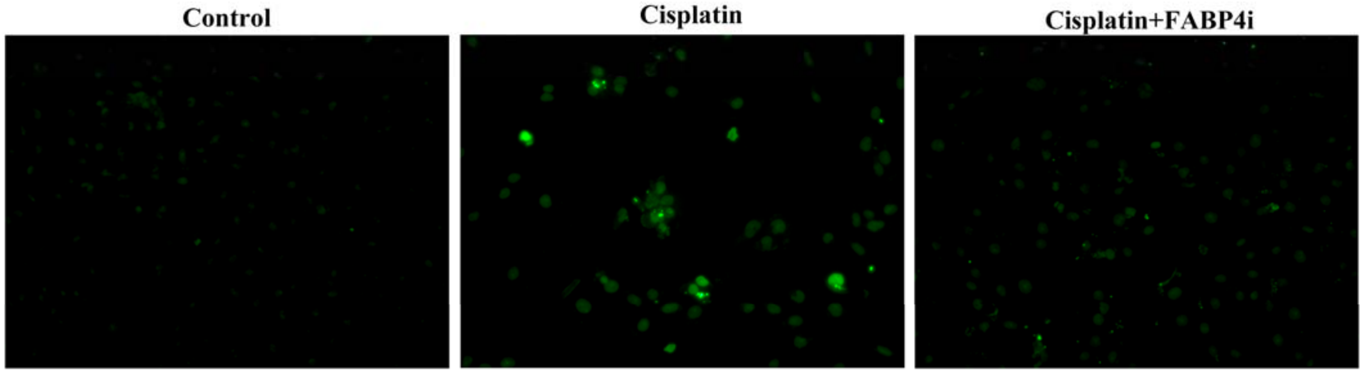

Figure S8. TUNEL staining of cisplatin-stimulated HK-2 cells. HK-2 cells were incubated with FABP4i BMS309403 at 10 μM 30 min prior to cisplatin treatment (20 μg/ml) for 24 h.
