# Supplementary material for: Pharmacological and genetic inhibition of fatty acid‐binding protein 4 alleviated cisplatin‐induced acute kidney injury
Source: J Cell Mol Med. 2019 Jul 8;23(9):6260–70. doi: 10.1111/jcmm.14512 (PMC6714212; doi:10.1111/jcmm.14512)

Table S1. Primer sequences

| Target | Forward | Reverse |
| --- | --- | --- |
| IL-1β | 5′-TGGGCCTCAAAGGAAAGAAT-3′ | 5′-CAGGCTTGTGCTCTGCTTGT-3′ |
| IL-6 | 5′-ACAACCACGGCCTTCCCTACTT-3′ | 5′-CACGATTTCCCAGAGAACATGTG-3′ |
| FABP4 | 5'-GGGGCCAGGCTTCTATTCC-3' | 5'-GGAGCTGGGTTAGGTATGGG-3' |
| KIM1 | 5′-ACATATCGTGGAATCACAACGAC-3′ | 5′-ACTGCTCTTCTGATAGGTGACA-3′ |
| NGAL | 5′-GCAGGTGGTACGTTGTGGG-3′ | 5′-CTCTTGTAGCTCATAGATGGTGC-3′ |
| GAPDH | 5′-GTATGACTCCACTCACGGCAAA-3′ | 5′-GGTCTCGCTCCTGGAAGATG-3′ |

Figure S1.


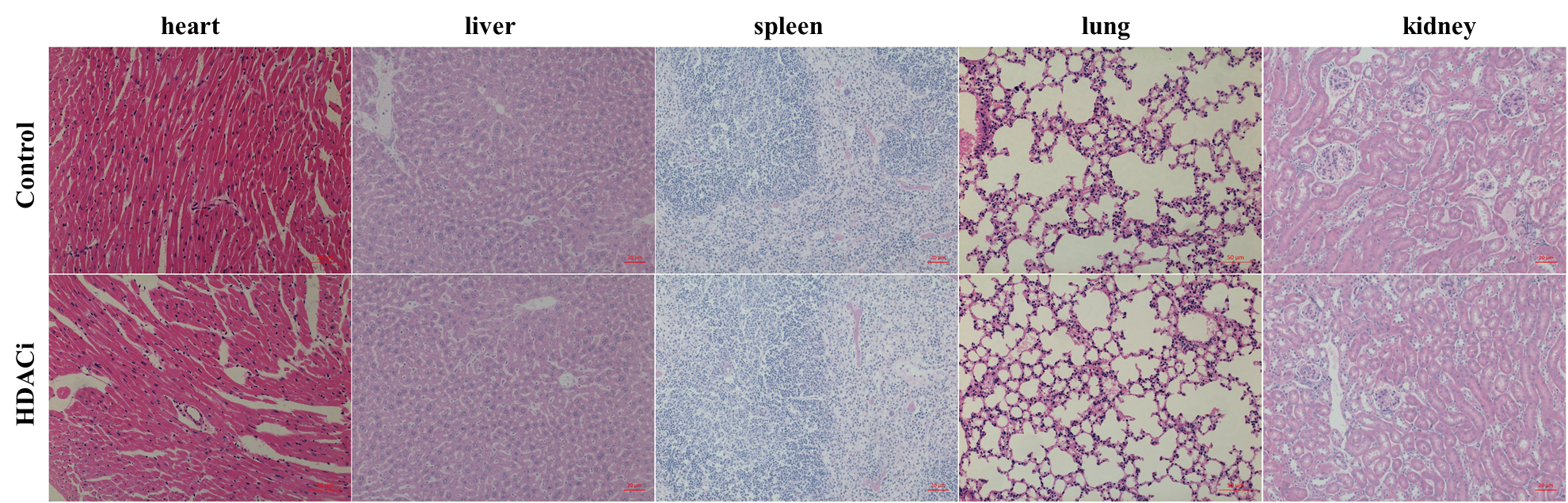


Figure S2.


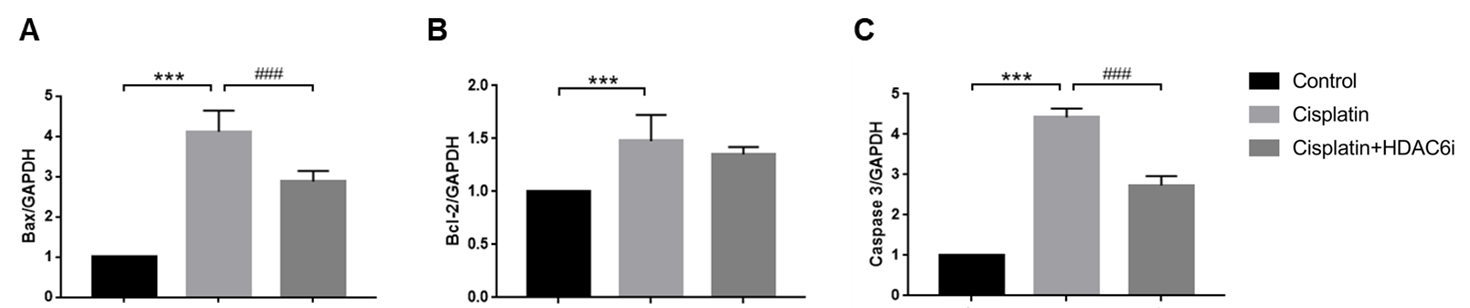


Figure S3.


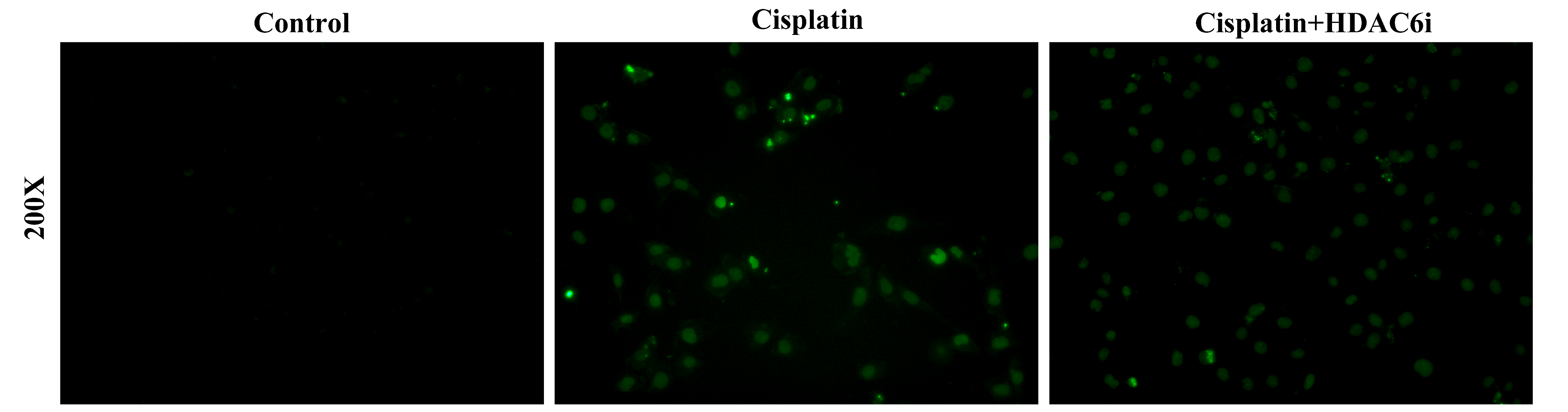

Supplement: Supplementary file 9 [file JCMM-23-6260-s009.doc]
