# Supplementary material for: Pharmacological and genetic inhibition of fatty acid‐binding protein 4 alleviated cisplatin‐induced acute kidney injury
Source: J Cell Mol Med. 2019 Jul 8;23(9):6260–70. doi: 10.1111/jcmm.14512 (PMC6714212; doi:10.1111/jcmm.14512)
Supplement: Supplementary file 10 [file JCMM-23-6260-s010.doc]

**Figure captions in supporting information**

**Figure S1. FABP4 knockout mice.** The design (A), target sequences (B) and identification (C) of FABP4 knockout mice.

**Figure S2. No pathologic changes of FABP4i BMS309403 on Heart, Liver, Spleen, Lung and Kidney tissues**. FABP4i was orally administrated to C57BL/6J mice at a dose of 40 mg/kg/d for 3 days.

**Figure S3. Densitometry values of apoptosis-related proteins in FABP4i-treated AKI mice.** The kidneys were taken for immunoblot analysis as shown in Figure 3. The densitometry values of proteins were normalized with GAPDH. Data expressed as means ± SD for groups of 3 independent experiments. *** P < 0.001; ** P < 0.01.

**Figure S4. Densitometry values of ER stress-related proteins in FABP4i-treated AKI mice.** The kidneys were taken for immunoblot analysis as shown in Figure 4. The densitometry values of proteins were normalized with GAPDH. Data expressed as means ± SD for groups of 3 independent experiments. *** P < 0.001; ** or ##P < 0.01; * or #P < 0.05.

**Figure S5. The mRNA expression of IL-1β and IL-6 in the kidneys of cisplatin-induced AKI**. FABP4i was orally administrated to C57BL/6J mice at a dose of 40 mg/kg/d for 3 days. All data are represented as the means±SE (n=6). **P<0.01, ***P<0.001.

**Figure S6. Densitometry values of ER stress-related proteins in FABP4 KO AKI mice.** The kidneys were taken for immunoblot analysis as shown in Figure 5. The densitometry values of proteins were normalized with GAPDH. Data expressed as means ± SD for groups of 3 independent experiments. *** or ### P < 0.001; ** or ##P < 0.01; * or #P < 0.05.

**Figure S7. Densitometry values of proteins in HK-2 cells.** The cells were taken for immunoblot analysis as shown in Figure 6. The densitometry values of proteins were normalized with GAPDH. Data expressed as means ± SD for groups of 3 independent experiments. *** or ### P < 0.001; ** or ##P < 0.01; #P < 0.05.

**Figure S8. TUNEL staining of cisplatin-stimulated HK-2 cells**. HK-2 cells were incubated with FABP4i BMS309403 at 10 μM 30 min prior to cisplatin treatment (20 μg/ml) for 24 h.
